# *Monkeypox Virus* Infection Resulting from an Occupational Needlestick — Florida, 2022

**DOI:** 10.15585/mmwr.mm7142e2

**Published:** 2022-10-21

**Authors:** Rafael Mendoza, Julia K. Petras, Patrick Jenkins, Margaret J. Gorensek, Susan Mableson, Philip A. Lee, Ann Carpenter, Heather Jones, Marie A. de Perio, Zeshan Chisty, Scott Brueck, Agam K. Rao, Johanna S. Salzer, Danielle Stanek, Carina Blackmore

**Affiliations:** ^1^Florida Department of Health in Broward County, Fort Lauderdale, Florida; ^2^CDC Monkeypox Emergency Response Team; ^3^Epidemic Intelligence Service, CDC; ^4^Holy Cross Health, Fort Lauderdale, Florida; ^5^Florida Department of Health.

In August 2022, the Florida Department of Health notified CDC of a nurse who acquired monkeypox through an occupational exposure while providing care to a patient with monkeypox. To date, occupationally acquired *Monkeypox virus* (MPXV) infections in health care personnel (HCP) have been rarely reported during the 2022 multinational outbreak (*1*,*2*). This report describes the first reported U.S. case and recommends approaches for preventing occupationally acquired MPXV infections in HCP.

On July 12, 2022, a Florida county health department (HD) received notification of an emergency department nurse who was exposed to MPXV through a needlestick that occurred earlier that day. While obtaining swabs from a patient with suspected monkeypox, the nurse used a needle to create an opening in the vesicular lesion to facilitate direct contact of the swab with fluid in the lesion. The needlestick occurred when recapping the used needle by hand before disposal; it caused a break in the skin on the index finger through the nurse’s gloved hand, accompanied by a small amount of bleeding. The wound was immediately washed with soap and water and drenched with Betadine antiseptic solution (10% povidone-iodine). The incident was promptly reported to the hospital’s infection control practitioner and occupational health department, and to the county HD. Later that day, the lesion swab collected from the patient by the nurse tested positive for nonvariola *Orthopoxvirus* using a real-time polymerase chain reaction (PCR) assay at the Florida Department of Health Bureau of Public Health Laboratories reference laboratory; a duplicate swab subsequently tested positive for Clade II (previously known as West African clade) MPXV at CDC using a real-time PCR assay specific for the detection of West African Clade II MPXV.

Within approximately 15 hours of the incident, the nurse, who had no relevant past medical history or previous orthopoxvirus vaccination, received the first dose of a 2-dose JYNNEOS vaccination series as postexposure prophylaxis. In accordance with CDC guidance (*3*), the nurse continued to work while asymptomatic and was actively monitored by the hospital infectious disease specialist and the county HD. The nurse wore a surgical mask, consistent with CDC COVID-19 guidance, and chose to wear medical gloves when interacting with patients.^†^

Ten days after the exposure, a single skin lesion formed at the site of the needlestick. The nurse immediately began isolating at home and kept the lesion covered until it had crusted over, the scab had fallen off, and a new layer of skin had formed beneath the lesion 19 days later.

The day after the single small vesicular lesion appeared, it was swabbed and subsequently tested positive by PCR for *Orthopoxvirus* and MPXV at a commercial laboratory; a duplicate swab tested at the Florida Department of Health Bureau of Public Health Laboratories reference laboratory using PCR was positive for nonvariola *Orthopoxvirus*. During the next 19 days, the lesion at the needlestick site increased in size (remaining <1 cm in diameter) and became pruritic, deep-seated, and umbilicated, then scabbed over and a new layer of skin formed under the scab. Apart from this single lesion at the puncture site, no additional lesions or other clinical signs or symptoms were reported, and tecovirimat was not indicated.^§^ No secondary cases were identified.

This report describes the first occupationally acquired MPVX infection in a U.S. health care worker during the 2022 monkeypox outbreak. CDC advises against unroofing, opening, or aspirating^¶^ (*4*) monkeypox lesions with sharp instruments (e.g., needles) and recapping used needles** because of the risk for sharps injuries. During the current outbreak, MPXV PCR testing cycle threshold values from swabbed skin and mucosal lesion specimens have been very low, indicating that surface swabbing collects sufficient amounts of viral material without a need to unroof lesions. Because of the reliability and sensitivity of real-time PCR assays used (*4*,*5*), vigorous swabbing of the outer surface of a lesion is adequate to collect enough viral material for testing and will minimize the potential for needlesticks. Employers should ensure that HCP are trained in proper specimen collection methods, follow recommended infection prevention and control precautions for the care of patients with monkeypox, and implement safety practices for managing sharps^††^ if they are used during other aspects of patient care. HCP with exposures should be evaluated promptly to ensure postexposure recommendations are implemented (*3*). As of October 6, 2022, among 326^§§^ HCP in Florida who have been occupationally exposed to patients with monkeypox during the 2022 outbreak, only this HCP with a reported needlestick exposure developed a clinical MPXV infection. Overall, with routine adherence to standard infection control practices, among U.S. HCP with nonpercutaneous exposure to monkeypox patients, the risk for acquiring monkeypox appears to be low (*6*).

## Footnotes

† A mask was worn consistent with CDC's health care guidance for the COVID-19 pandemic. https://www.cdc.gov/coronavirus/2019-ncov/hcp/infection-control-recommendations.html (Accessed October 14, 2022.) CDC does not recommend work restriction or use of additional personal protective equipment for asymptomatic HCP while being monitored for symptoms compatible with monkeypox.

§
https://www.cdc.gov/poxvirus/monkeypox/clinicians/Tecovirimat.html

¶
https://www.cdc.gov/poxvirus/monkeypox/clinicians/prep-collection-specimens.html

**
https://www.cdc.gov/niosh/newsroom/feature/needlestick_disposal.html

††
https://www.cdc.gov/sharpssafety/index.html

§§ Thirty-one of these 326 exposures were determined to be high risk, 47 intermediate risk, and 248 low or uncertain risk using the exposure criteria in effect at the time of risk assessment. http://web.archive.org/web/20220706182701/https:/www.cdc.gov/poxvirus/monkeypox/clinicians/monitoring.html
